# Comparison of Deep Tissue Massage and Therapeutic Massage for Lower Back Pain, Disease Activity, and Functional Capacity of Ankylosing Spondylitis Patients: A Randomized Clinical Pilot Study

**DOI:** 10.1155/2017/9894128

**Published:** 2017-08-06

**Authors:** Mateusz Wojciech Romanowski, Maja Špiritović, Radosław Rutkowski, Adrian Dudek, Włodzimierz Samborski, Anna Straburzyńska-Lupa

**Affiliations:** ^1^Department of Rheumatology and Rehabilitation, Poznan University of Medical Sciences, Poznan, Poland; ^2^Rheumatological Centre, Śrem, Poland; ^3^Institute of Rheumatology and Clinic of Rheumatology, Charles University, Prague, Czech Republic; ^4^Department of Physical Therapy and Sports Recovery, Poznan University of Physical Education, Poznan, Poland

## Abstract

**Objectives:**

This study aims to compare the effectiveness of deep tissue massage (DTM) and therapeutic massage (TM) in the management of ankylosing spondylitis (AS) patients.

**Materials and Methods:**

This was a small, randomized clinical pilot study. Subjects were 27 men with diagnosed AS, randomly assigned to DTM group or TM group. Subjects in each group had 10 sessions of massage. Outcomes included the Bath Ankylosing Spondylitis Disease Activity Index (BASDAI), the Bath Ankylosing Spondylitis Functional Index (BASFI), Modified Schober Test, Finger to Floor Test, chest expansion, and pain intensity of lower back.

**Results:**

There are no statistical significant differences between groups, except for BASDAI and pain intensity of lower back.

**Conclusions:**

This study suggests that massage may have clinical benefits for treating ankylosing spondylitis patients. Additional scientific research in this area is warranted.

## 1. Introduction

Ankylosing spondylitis (AS) is a chronic, progressive inflammatory rheumatic disease that predominantly affects the sacroiliac joints and spine and it may also involve the peripheral joints and specific organ like the eyes and bowels. AS leads to structural damage and functional impairments and a decrease in the quality of life [1]. The majority of AS patients suffer from back pain [2]. Lower back pain and stiffness are clinical criteria for the diagnosis of AS [3].

AS affects 0.1–0.5% of the population in Central Europe [2]. The latest publications indicate that spondyloarthropathies occur as often as rheumatoid arthritis [4, 5]. Men are affected by AS 2-3 times more often than women [1]. In 80% of cases, the first symptoms of AS occur in people under the age of 30 and only in 5% of patients over 45 years of age [3].

The treatment of AS patients requires pharmacotherapy together with nonpharmacological intervention, including physiotherapy modalities [6, 7]. The relevant physiotherapy modalities in the management of AS include supervised and unsupervised exercises, training, manual therapy, massage, hydrotherapy, electrotherapy, acupuncture, patient information and educational programs [8], balneotherapy, spa therapy, rehabilitation [9], education [10], and lifestyle modification [11]. Wang et al. [12] reported that the home exercise program is the most convenient and thus the first choice for patients with AS.

The main goals of therapy are to reduce pain, prevent spinal deformation, and improve mobility and overall function [7]. Physiotherapy is considered to play an important role in AS management and helps to maintain functional abilities and a satisfactory quality of life [7, 8]. However, the optimal management of AS still remains unresolved [13].

The Ottawa Panel believes that massage is an effective therapy in helping to relive subacute and chronic lower back pain (LBP) [14]. Furthermore, the Philadelphia Panel selected massage as rehabilitation interventions to formulate evidence-based practice guidelines (EBPGs) for the management of the lower back, neck, knee, and shoulder pain [15]. The Cochrane Review stated that the benefits of massage for patients with acute, subacute, and chronic nonspecific LBP were found mostly in the short-term follow-up period (up to six months after randomization) for pain outcomes [16].

However only a few studies, mostly case studies, have discussed the effect of massage therapy on LBP in AS patients [17, 18].

The purpose of this study is to compare the effectiveness of deep tissue massage and therapeutic massage in the management of AS patients.

## 2. Materials and Methods

### 2.1. Study Design

The study was a randomized clinical pilot study with unblinded treatment and blinded outcome assessment. It included eligible patients who were recruited from the patients who were admitted to the rheumatology department at this time with a diagnosis of AS according to the modified New York criteria [2].

After baseline assessments, 31 male patients were randomly assigned to Group DTM (deep tissue massage) and Group TM (therapeutic massage). Patients were randomized to DTM or TM using sealed opaque envelopes indicating treatment allocation. Randomization envelopes were prepared at study inception and random number sequence was obtained by flipping a coin. A research assistant not involved in the conduct of the study randomized patients, allocated treatment, and collected key data. Exclusion and inclusion criteria for this study are shown in Table 1.

All procedures performed in studies involving human participants were in accordance with the ethical standards of the institutional and/or national research committee and with the 1964 Helsinki declaration and its later amendments on comparable ethical standards. Informed consent was obtained from all individual participants included in the study.

This study was approved by the Bioethics Committee of the University of Medical Sciences in Poznan (trial number 645/15).

### 2.2. Interventions

#### 2.2.1. Patients from Both Groups

 Patients from both groups, DTM (deep tissue massage) and TM (therapeutic massage), underwent a 30-minute session of deep tissue massage or therapeutic massage daily for 2 weeks (a total of 10 sessions). The massage was provided by 2 licensed therapists with at least 5 years of experience who were comfortable following the study protocol and had experience in the permitted techniques. Drugs prescribed earlier by their specialist were used in a consistent dosage.

#### 2.2.2. Deep Tissue Massage

The massage therapist gave a series of 10 back massages intended to identify and alleviate musculoskeletal contributors to the participants' lower back pain. It was performed by using trigger point therapy and oblique pressure for a combination of lengthening strokes (extending a particular joint while at the same time working the muscle in the direction of the lengthening), cross-fiber strokes (rolling the fingers over the tendon or muscle, back and forth, perpendicular to the fiber direction for two or three minutes), anchor and stretch (anchoring at a tight area and stretching away from the spot), and freeing muscle from entrapment (mobilizing the erector spinae muscle in the lateral/medial direction by using both fingers of both hands to apply force along the border of the muscle and slowly push the muscle towards the opposite side).

#### 2.2.3. Therapeutic Massage

The massage therapist gave a series of 10 back massages (from sacrum to occipital bone) intended to ease lower back pain and improve function by inducing a generalized sense of relaxation. Five distinct techniques were permitted: effleurage (gliding), petrissage (kneading, rolling), friction, holding, and vibration.

### 2.3. Primary Outcome Measure

The Bath Ankylosing Spondylitis Disease Activity Index (BASDAI) consisted of 6 questions using a numerical rating scale (0–10) to measure the severity of fatigue, spinal and peripheral joint pain, localised tenderness, and morning stiffness (both qualitative and quantitative) [19].

The Bath Ankylosing Spondylitis Functional Index (BASFI) consists of 8 specific questions regarding function in AS and 2 questions reflecting the patient's ability to cope with everyday life. Each question was answered on a numerical rating scale (0–10). The mean score of the 10 items then provided the final BASFI score [19].

### 2.4. Secondary Outcome Measures

Spinal mobility was assessed by the Modified Schober (MS) Test and Finger to Floor (FTF) Test. MS increase in the distance between two skin marks 10 cm above and 5 cm below the connecting line between “the Dimples of Venus” after maximal forward bending, measured with a tape. FTF is the distance between fingertips and floor measured with tape at maximal flexion of spine and pelvis while the knees are kept in extension [19].

Chest expansion (CE) is defined as the difference in chest circumference at maximal inspiration and expiration at the level of the fourth intercostal space [19].

Pain intensity was assessed by Visual Analog Scale (VAS). Participants were asked to place a line perpendicular to the VAS line at the point that represents their lower back pain during the past week anchored by “no pain” (score of 0) and “most severe pain” (score of 100 [100 mm scale]) [19].

### 2.5. Statistical Analyses

For demographic data, independent *t*-test or Mann–Whitney *U* test (if distribution was nonnormal) was used for continuous variable to check difference between the DTM and the TM groups in baseline data. The normal distribution of each group was checked by the Shapiro-Wilk test. For each participant, change scores were calculated by subtracting the result of the final from those at baseline. In the study, mean change scores within groups and differences in change scores between groups were calculated, with 95% confidence intervals. Statistical analyses were performed using SPSS 24.0 for Windows. A *p* value of <0.05 was regarded as significant.

## 3. Results

**Figure 1:**
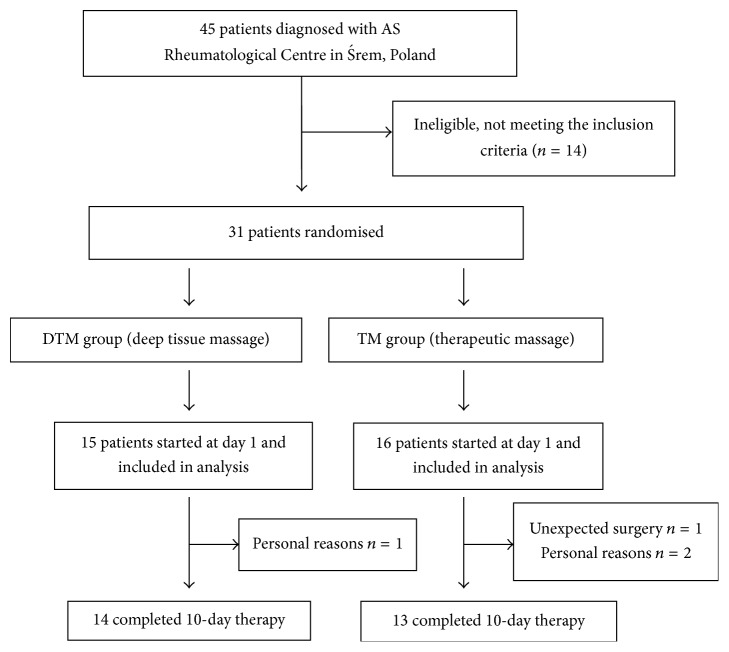
Trial profile.

The flow of the participants through the trial is shown in Figure 1(). Based on the exclusion and inclusion criteria, 31 patients from 45 were included in this trial. For unexpected surgical and personal reason 4 more patients were excluded from this study.

Patients from the Group DTM and the Group TM did not differ in basic characteristics before therapy in terms of age, BMI, ESR, CRP, BMI, BASDAI, BASFI, CE, MS, FTF, and PAIN (Table 2).

### 3.1. Primary Outcome Measure

After intervention the DTM group showed a significantly greater reduction in BASDAI than the TM group (Table 3). Effect sizes were large. There were no significant differences in BASFI between the groups.

### 3.2. Secondary Outcome Measures

After intervention the DTM group showed a significantly greater reduction in pain than the TM group (Table 3). Effect sizes were large. There were no significant differences in Modified Schober Test, the distance between fingertips and floor, and chest expansion between the groups.

## 4. Discussion

This was a small, randomized clinical pilot study to compare deep tissue massage and therapeutic massage on lower back pain, disease activity, and the functional capacity of AS patients. A review of the available literature did not reveal any similar studies on AS patients. Regarding the role of physiotherapy in AS, research has mainly focused on exercise therapy and little has been documented on the other physical therapy modalities [20].

Our results showed there are no statistical significant differences between groups, except for BASDAI and PAIN.

The management of ankylosing spondylitis based on current evidence does not include massage therapy as an effective treatment for AS patient [6, 9, 11]. Massage is mentioned as a possible treatment in some studies but without any research to confirm its effectiveness [7, 10, 21].

Individual selection of therapy in ankylosing spondylitis is extremely important. Based on the rules for the implementation of deep massage, we can better interact with the patient. First of all, deep tissue massage is not a repeated sequence of successive movements. A therapist using the techniques of deep tissue massage should pay attention to how the tissue reacts to the treatment and continually analyses and reaches its loosening, so DTM is performed with force that is individually matched to the patient and depends on the depth which the tissue requires. Minor forces will be used when working on superficial fascia and greater force should be used on the back extensor. The angle between the massaged tissue and therapist's hand is also important, and it should not be greater than 45 degrees to achieve the best tissue relaxing effect [18]. Possible mechanisms of massage are biomechanical (mechanical pressure on tissues), physiological (changes in tissue or organ), neurological (reflex stimulation), and psychological (increased relationship between body and mind) [22, 23].

Massage therapist performing DTM of patient with AS must pay particular attention to the correct position of the patient, which should be comfortable and fully relaxed. Therefore, therapist may choose between supine and side position. DTM should be performed slowly and its goal is to achieve relaxation of soft tissue. Very important are palpation skills of the therapist, which will allow locating strained muscle fibers and trigger point and be let for specific work in these places. DTM will help reducing restrictive barriers or fibrous adhesion seen between layers of fascial tissue.

In the literature we found 4 articles where massage therapy was used to treat an AS patient. Massage described as passive treatment was used in the research by Strumse et al. [24]. It is difficult to conclude if massage was effective or not, because it was just a possible option for the patient. Two passive treatments, from a possible three, lasting for 10–15 min a day were usually given to each patient. The patients received passive therapy only when this was indicated by the physiotherapist. Occasionally, massage could be added to a supervised physiotherapy group for 1 hour, 5 days per week, 3 weeks + hydrotherapy 3 times per week in the research by Helliwell et al. [25]. However, the use of massage was not taken into consideration in formulating the final conclusions [8].

In the literature we found two case studies using massage therapy in AS patients. Massage reduces back pain, stiffness, and fatigue and could be used as a complement for the standard care of people with AS [17]. The reduction in back pain is in line with our findings. In this study we did not particularly focus on stiffness and fatigue but those components are parts of the BASDAI which we did measure and both therapeutic and deep tissue massage decreased BASDAI. Romanowski and Straburzyńska-Lupa [18] concluded that 10 sessions of deep tissue massage lead to improvement in functional capacity (BASFI) and spinal mobility improvement. We confirm this in our study.

Massage decreased pain for acute, subacute, and chronic nonspecific LBP when compared with inactive and active controls mostly in the short-term follow-up period [16]. Furlan et al. [26] reported that massage was effective in reducing the intensity of pain and in improving functionality in patients with chronic lumbosacral pain. There are a small number of papers on deep tissue massage which show the effectiveness of this form of massage in the treatment of lower back pain. Romanowski et al. [27] indicated greater effectiveness of DTM in comparison with therapeutic massage with regard to a patient's pain sensation. DTM may help to reduce the use of NSAID in the treatment of chronic lower back pain [28]. DTM decreases low back pain and improves functional capacity of pregnant women [29]. Studies show correlation between DTM and reduction in blood pressure and heart rate [30].

There is a need for meta-analysis of larger and better studies with more specific populations, interventions, cointerventions, and outcome measures.

This study is the first randomized clinical pilot study comparing the effectiveness of deep tissue massage and therapeutic massage on lower back pain in AS patients. An attempt was made to check the effects of deep tissue massage and therapeutic massage on the possible reduction of the intensity of lower back pain, decrease in disease activity, and improvement in functional capacity. Our study suggests that the use of deep tissue massage and therapeutic massage might have therapeutic results and that supplementing the comprehensive rehabilitation of an AS patient could be considered for use. The results obtained due to the small size of the groups do not allow for final conclusion about the role of massage in the treatment of AS but are very encouraging and can stimulate further studies in this field.

## Figures and Tables

**Table 1:** Criteria for inclusion and exclusion.

Inclusion in the study	Exclusion from the study
Diagnosis of AS Age range: 20–60 years Informed consent for participation in the study, signed by the patient	Change in dosage of nonsteroidal anti-inflammatory drugs and corticosteroids for 2 weeks before the beginning of the study and for the duration of the study Change in dosage of the disease-modifying synthetic and biologics antirheumatic drugs for 3 months before the beginning of the study and for the duration of the study Injection of a local anesthetic, steroids for 1 months before the beginning of the study and for the duration of the study Surgery, recently, in the past 6 months Neurological signs present Cancer Discitis Disk disease Fracture of vertebra Infectious cause of back pain Scoliosis, severe, or progressive Spinal stenosis Spondylolisthesis Fever

**Table 2:** Baseline characteristics of the 27 patients with ankylosing spondylitis.

	DTM group (*n* = 14)	TM group (*n* = 13)	*p*
Age (years)	49 (7.78)	45 (6.71)	0.351
ESR (mm/h)	26.1 (8.07)	27.3 (11.71)	0.758
CRP (mg/l)	27.0 (9.41)	28.9 (12.56)	0.418
BMI	27.9 (2.63)	31.4 (11.64)	0.394
BASDAI [NRS]	6.9 (1.45)	6.7 (0.89)	0.763
BASFI [NRS]	6.2 (1.23)	5.7 (1.56)	0.274
CE [cm]	1.6 (0.49)	1.3 (0.81)	0.188
MS [cm]	1.5 (0.83)	1.7 (1.44)	0.380
FTF [cm]	20.56 (9.96)	19.23 (7.39)	0.460
PAIN [VAS]	69.8 (12.51)	63.3 (10.49)	0.107

NSAID	12	11	
DMARD synthetic	9	8	
DMARD biologics	1	1	

The results are expressed as mean (SD); BMI: body mass index.; BASDAI: Bath Ankylosing Spondylitis Disease Activity Index; BASFI: Bath Ankylosing Spondylitis Functional Index; CE: chest expansion; MS: Modified Schober; FTF: Finger to Floor; PAIN: intensity of lower back pain; NRS: numerical rating scale; VAS: Visual Analog Scale; NSAID: nonsteroidal anti-inflammatory drugs; DMARDs: disease-modifying antirheumatic drugs; *p* values for differences in the baseline data between the DTM and the TM groups.

**Table 3:** Mean score differences over time according to group allocation using intention to treat analysis.

	DTM group (*n* = 14) deep tissue massage mean (95% CI)	TM group (*n* = 13) therapeutic massage mean (95% CI)	The difference in change between the groups mean (95% CI)	Effect size	Both groups
	Change in DTM group	Change in TM group			*p*
BASDAI [NRS]	−1.21 (−1.82 to −0.61)	−0.68 (−0.84 to −0.53)	−0.53 (−1.14 to 0.08)	0.69	0.021
BASFI [NRS]	−0.50 (−0.88 to −0,12)	−0.35 (−0.49 to −0.20)	−0.15 (−0.56 to 0.26)	0.31	0.896
CE [cm]	0.32 (0.11 to 0.54)	0.31 (0.08 to 0.54)	0.01 (−0.29 to 0.31)	0.03	0.915
MS [cm]	0.57 (0.47 to 0.68)	0.50 (0.25 to 0.75)	0.07 (−0.16 to 0.30)	0.22	0.328
FTF [cm]	−2.57 (−3.11 to −2.03)	−2.19 (−2.53 to −1.85)	−0.38 (−0.99 to 0.24)	0.49	0.222
PAIN [VAS]	−35.21 (−45.19 to −25.23)	−15.23 (−22.20 to −8.25)	−19.98 (−31.74 to −8.23)	1.36	0.003

BASDAI: Bath Ankylosing Spondylitis Disease Activity Index; BASFI: Bath Ankylosing Spondylitis Functional Index; CE: chest expansion; MS: Modified Schober; FTF: Finger to Floor; PAIN: intensity of lower back pain; NRS: numerical rating scale; VAS: Visual Analog Scale; *p* values for differences in the changed score between the baseline and the posttherapy data between the DTM and the TM groups.

## Additional Points

*Study Limitations. *The lack of a control group with no massage in the present study does not allow us to make the conclusions on the usefulness of any kind of massage for AS patients as a self-care for pain relief.
